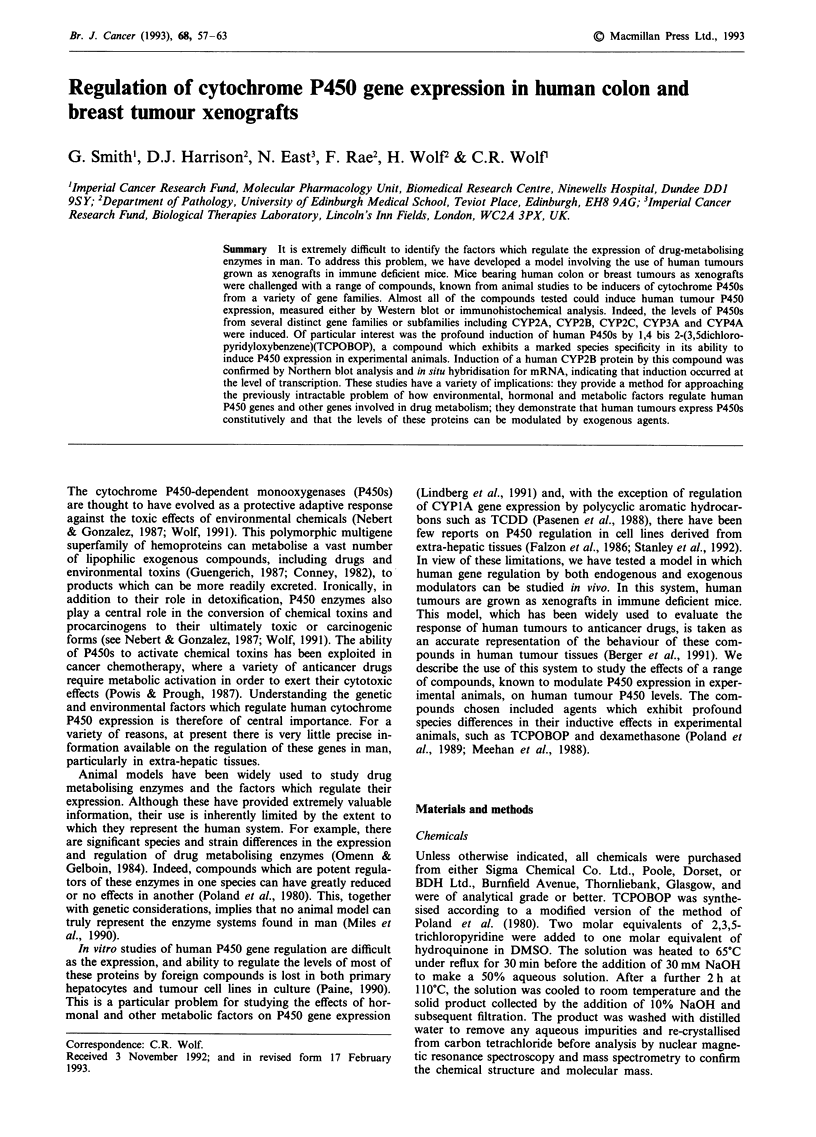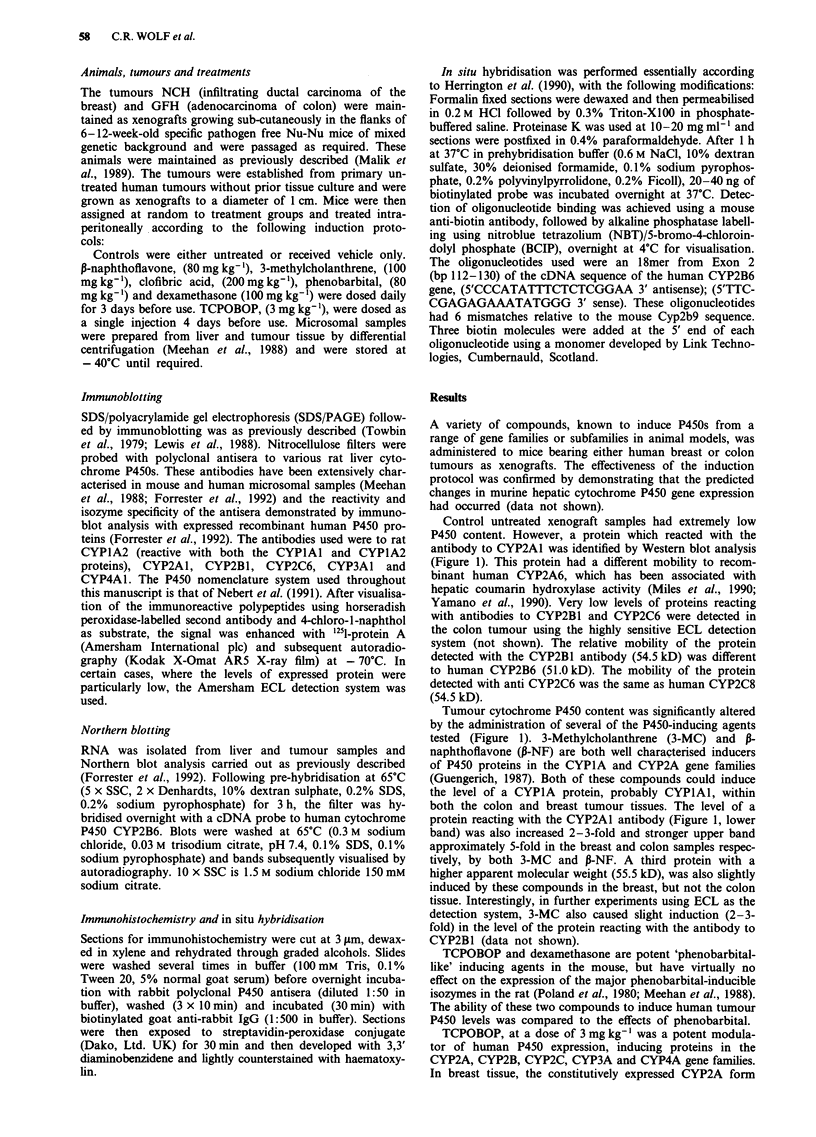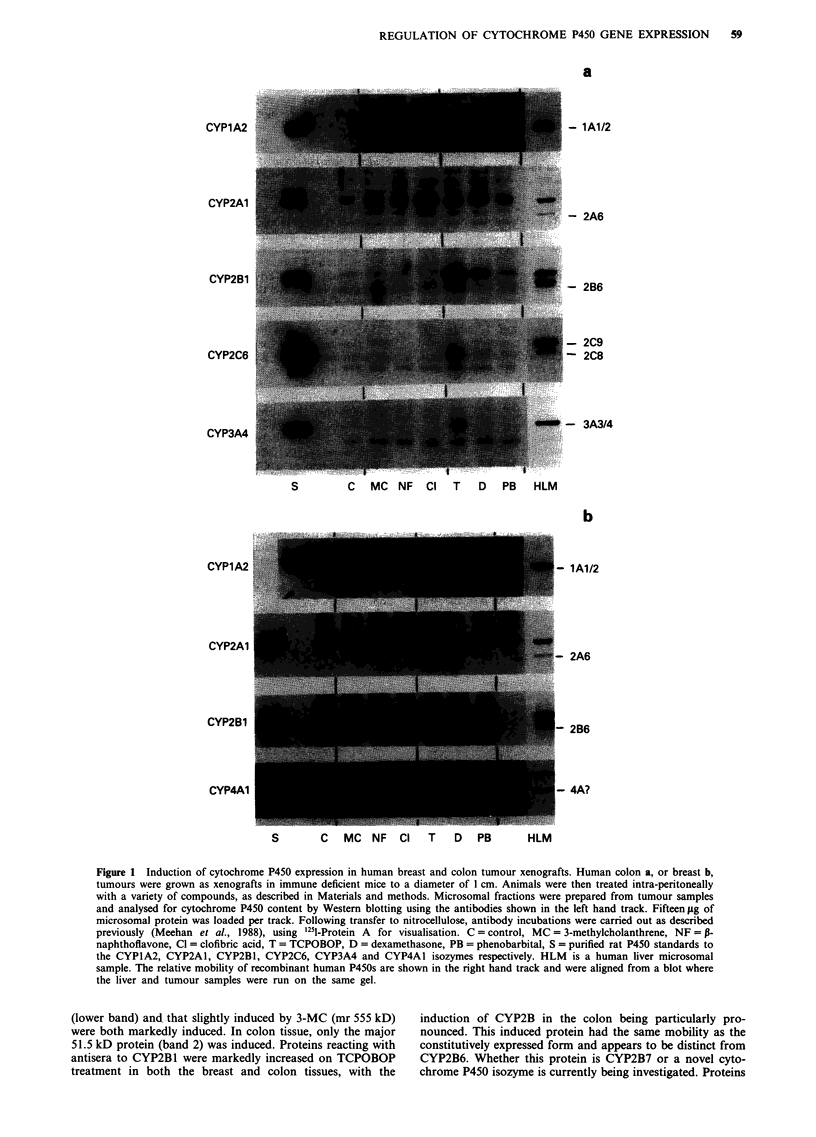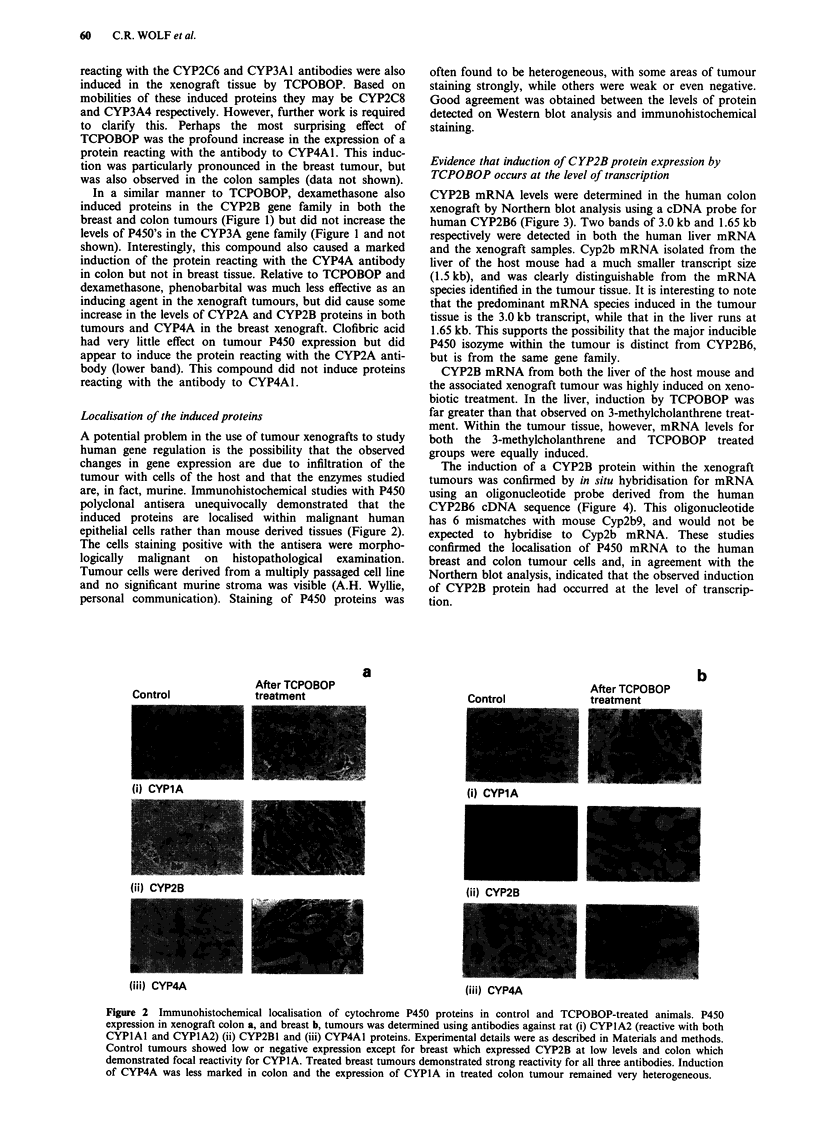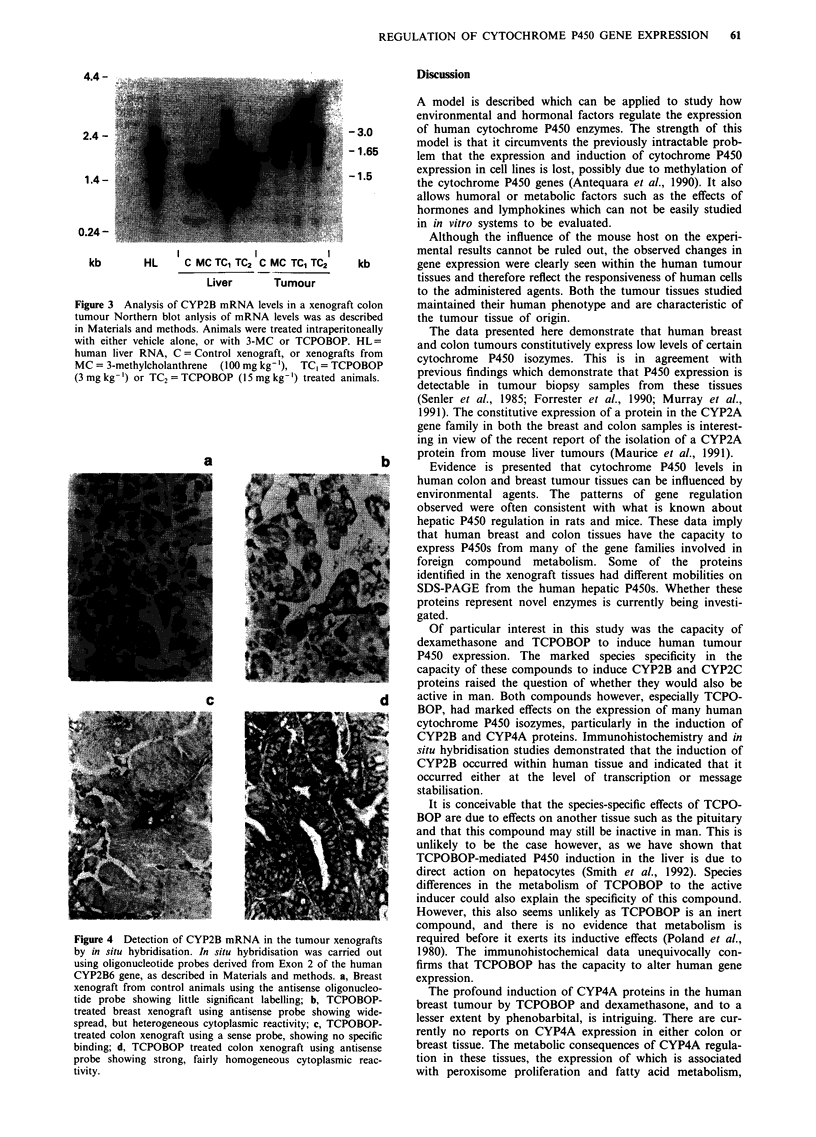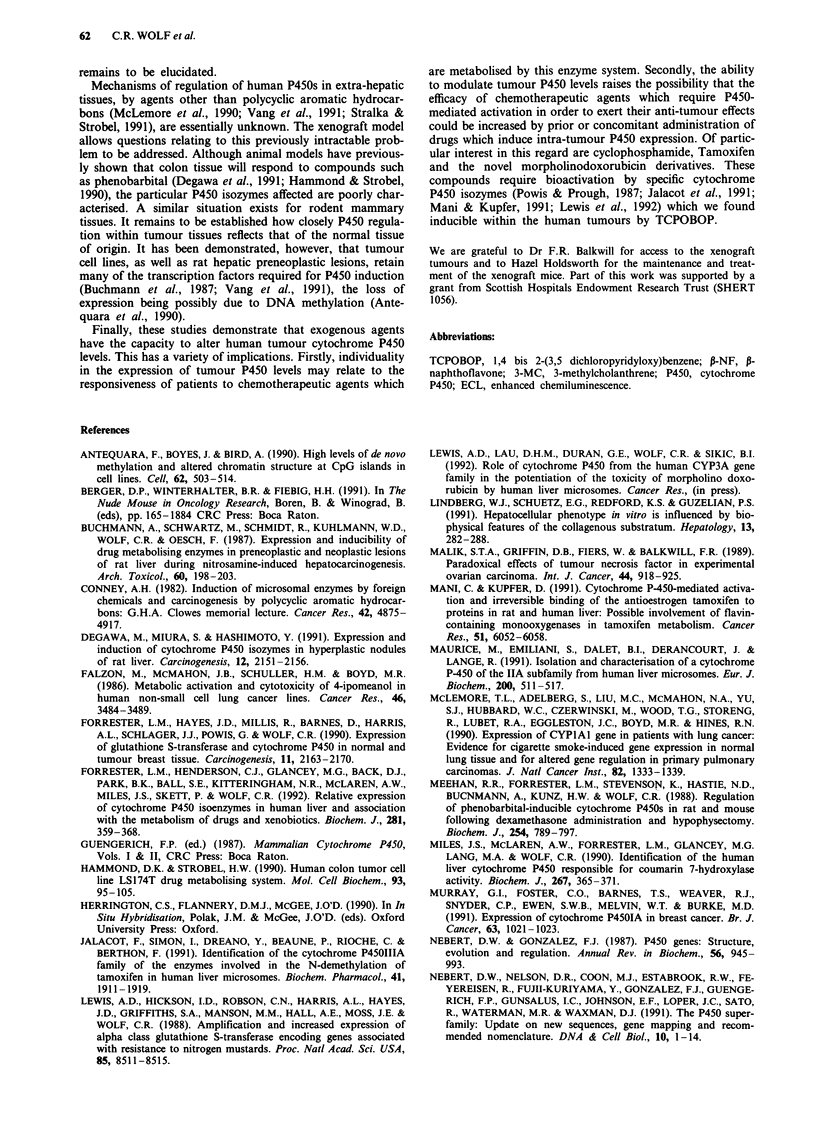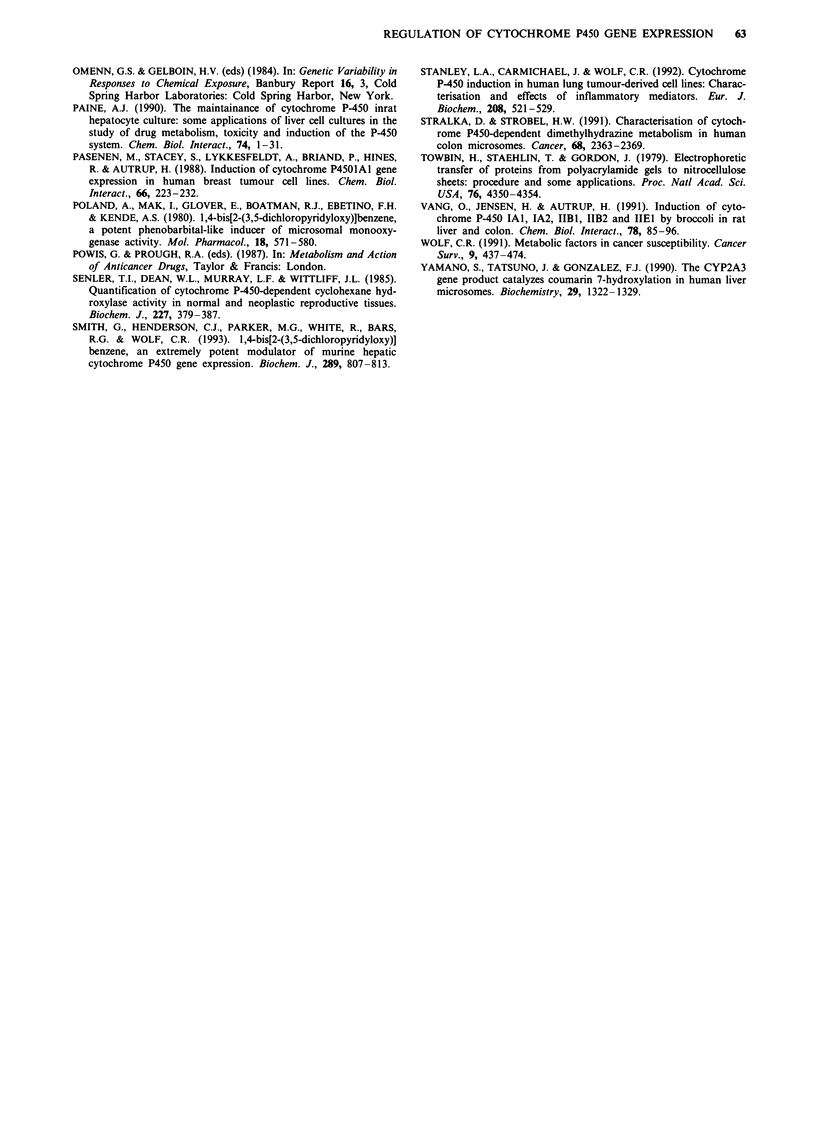# Regulation of cytochrome P450 gene expression in human colon and breast tumour xenografts.

**DOI:** 10.1038/bjc.1993.286

**Published:** 1993-07

**Authors:** G. Smith, D. J. Harrison, N. East, F. Rae, H. Wolf, C. R. Wolf

**Affiliations:** Imperial Cancer Research Fund, Molecular Pharmacology Unit, Ninewells Hospital, Dundee, UK.

## Abstract

**Images:**


Br. J. Cancer (1993), 68, 57-63 © Macmillan Press Ltd., 1993

### Regulation of cytochrome P450 gene expression in human colon and breast tumour xenografts

G. Smith[1], D.J. Harrison[2], N. East[3], F. Rae[2], H. Wolf[2] & C.R. Wolf[1]

[1]*Imperial Cancer Research Fund, Molecular Pharmacology Unit, Biomedical Research Centre, Ninewells Hospital, Dundee DD1 9SY;* [2]*Department of Pathology, University of Edinburgh Medical School, Teviot Place, Edinburgh, EH8 9AG;* [3]*Imperial Cancer Research Fund, Biological Therapies Laboratory, Lincoln's Inn Fields, London, WC2A 3PX, UK.*

**Summary** It is extremely difficult to identify the factors which regulate the expression of drug-metabolising enzymes in man. To address this problem, we have developed a model involving the use of human tumours grown as xenografts in immune deficient mice. Mice bearing human colon or breast tumours as xenografts were challenged with a range of compounds, known from animal studies to be inducers of cytochrome P450s from a variety of gene families. Almost all of the compounds tested could induce human tumour P450 expression, measured either by Western blot or immunohistochemical analysis. Indeed, the levels of P450s from several distinct gene families or subfamilies including CYP2A, CYP2B, CYP2C, CYP3A and CYP4A were induced. Of particular interest was the profound induction of human P450s by 1,4 bis 2-(3,5dichloro-pyridyloxybenzene)(TCPOBOP), a compound which exhibits a marked species specificity in its ability to induce P450 expression in experimental animals. Induction of a human CYP2B protein by this compound was confirmed by Northern blot analysis and *in situ* hybridisation for mRNA, indicating that induction occurred at the level of transcription. These studies have a variety of implications: they provide a method for approaching the previously intractable problem of how environmental, hormonal and metabolic factors regulate human P450 genes and other genes involved in drug metabolism; they demonstrate that human tumours express P450s constitutively and that the levels of these proteins can be modulated by exogenous agents.

The cytochrome P450-dependent monooxygenases (P450s) are thought to have evolved as a protective adaptive response against the toxic effects of environmental chemicals (Nebert & Gonzalez, 1987; Wolf, 1991). This polymorphic multigene superfamily of hemoproteins can metabolise a vast number of lipophilic exogenous compounds, including drugs and environmental toxins (Guengerich, 1987; Conney, 1982), to products which can be more readily excreted. Ironically, in addition to their role in detoxification, P450 enzymes also play a central role in the conversion of chemical toxins and procarcinogens to their ultimately toxic or carcinogenic forms (see Nebert & Gonzalez, 1987; Wolf, 1991). The ability of P450s to activate chemical toxins has been exploited in cancer chemotherapy, where a variety of anticancer drugs require metabolic activation in order to exert their cytotoxic effects (Powis & Prough, 1987). Understanding the genetic and environmental factors which regulate human cytochrome P450 expression is therefore of central importance. For a variety of reasons, at present there is very little precise information available on the regulation of these genes in man, particularly in extra-hepatic tissues.

Animal models have been widely used to study drug metabolising enzymes and the factors which regulate their expression. Although these have provided extremely valuable information, their use is inherently limited by the extent to which they represent the human system. For example, there are significant species and strain differences in the expression and regulation of drug metabolising enzymes (Omenn & Gelboin, 1984). Indeed, compounds which are potent regulators of these enzymes in one species can have greatly reduced or no effects in another (Poland *et al.*, 1980). This, together with genetic considerations, implies that no animal model can truly represent the enzyme systems found in man (Miles *et al.*, 1990).

*In vitro* studies of human P450 gene regulation are difficult as the expression, and ability to regulate the levels of most of these proteins by foreign compounds is lost in both primary hepatocytes and tumour cell lines in culture (Paine, 1990). This is a particular problem for studying the effects of hormonal and other metabolic factors on P450 gene expression (Lindberg *et al.*, 1991) and, with the exception of regulation of CYP1A gene expression by polycyclic aromatic hydrocarbons such as TCDD (Pasenen *et al.*, 1988), there have been few reports on P450 regulation in cell lines derived from extra-hepatic tissues (Falzon *et al.*, 1986; Stanley *et al.*, 1992). In view of these limitations, we have tested a model in which human gene regulation by both endogenous and exogenous modulators can be studied *in vivo*. In this system, human tumours are grown as xenografts in immune deficient mice. This model, which has been widely used to evaluate the response of human tumours to anticancer drugs, is taken as an accurate representation of the behaviour of these compounds in human tumour tissues (Berger *et al.*, 1991). We describe the use of this system to study the effects of a range of compounds, known to modulate P450 expression in experimental animals, on human tumour P450 levels. The compounds chosen included agents which exhibit profound species differences in their inductive effects in experimental animals, such as TCPOBOP and dexamethasone (Poland *et al.*, 1989; Meehan *et al.*, 1988).

### Materials and methods

### *Chemicals*

Unless otherwise indicated, all chemicals were purchased from either Sigma Chemical Co. Ltd., Poole, Dorset, or BDH Ltd., Burnfield Avenue, Thornliebank, Glasgow, and were of analytical grade or better. TCPOBOP was synthesised according to a modified version of the method of Poland *et al.* (1980). Two molar equivalents of 2,3,5-trichloropyridine were added to one molar equivalent of hydroquinone in DMSO. The solution was heated to 65°C under reflux for 30 min before the addition of 30 mM NaOH to make a 50% aqueous solution. After a further 2 h at 110°C, the solution was cooled to room temperature and the solid product collected by the addition of 10% NaOH and subsequent filtration. The product was washed with distilled water to remove any aqueous impurities and re-crystallised from carbon tetrachloride before analysis by nuclear magnetic resonance spectroscopy and mass spectrometry to confirm the chemical structure and molecular mass.

Correspondence: C.R. Wolf.
Received 3 November 1992; and in revised form 17 February 1993.

*Animals, tumours and treatments*

The tumours NCH (infiltrating ductal carcinoma of the breast) and GFH (adenocarcinoma of colon) were maintained as xenografts growing sub-cutaneously in the flanks of 6–12-week-old specific pathogen free Nu-Nu mice of mixed genetic background and were passaged as required. These animals were maintained as previously described (Malik *et al.*, 1989). The tumours were established from primary untreated human tumours without prior tissue culture and were grown as xenografts to a diameter of 1 cm. Mice were then assigned at random to treatment groups and treated intraperitoneally according to the following induction protocols:

Controls were either untreated or received vehicle only. β-naphthoflavone, (80 mg kg$^{-1}$), 3-methylcholanthrene, (100 mg kg$^{-1}$), clofibric acid, (200 mg kg$^{-1}$), phenobarbital, (80 mg kg$^{-1}$) and dexamethasone (100 mg kg$^{-1}$) were dosed daily for 3 days before use. TCPOBOP, (3 mg kg$^{-1}$), were dosed as a single injection 4 days before use. Microsomal samples were prepared from liver and tumour tissue by differential centrifugation (Meehan *et al.*, 1988) and were stored at − 40°C until required.

*Immunoblotting*

SDS/polyacrylamide gel electrophoresis (SDS/PAGE) followed by immunoblotting was as previously described (Towbin *et al.*, 1979; Lewis *et al.*, 1988). Nitrocellulose filters were probed with polyclonal antisera to various rat liver cytochrome P450s. These antibodies have been extensively characterised in mouse and human microsomal samples (Meehan *et al.*, 1988; Forrester *et al.*, 1992) and the reactivity and isozyme specificity of the antisera demonstrated by immunoblot analysis with expressed recombinant human P450 proteins (Forrester *et al.*, 1992). The antibodies used were to rat CYP1A2 (reactive with both the CYP1A1 and CYP1A2 proteins), CYP2A1, CYP2B1, CYP2C6, CYP3A1 and CYP4A1. The P450 nomenclature system used throughout this manuscript is that of Nebert *et al.* (1991). After visualisation of the immunoreactive polypeptides using horseradish peroxidase-labelled second antibody and 4-chloro-1-naphthol as substrate, the signal was enhanced with $^{125}$I-protein A (Amersham International plc) and subsequent autoradiography (Kodak X-Omat AR5 X-ray film) at − 70°C. In certain cases, where the levels of expressed protein were particularly low, the Amersham ECL detection system was used.

*Northern blotting*

RNA was isolated from liver and tumour samples and Northern blot analysis carried out as previously described (Forrester *et al.*, 1992). Following pre-hybridisation at 65°C (5 × SSC, 2 × Denhardts, 10% dextran sulphate, 0.2% SDS, 0.2% sodium pyrophosphate) for 3 h, the filter was hybridised overnight with a cDNA probe to human cytochrome P450 CYP2B6. Blots were washed at 65°C (0.3 M sodium chloride, 0.03 M trisodium citrate, pH 7.4, 0.1% SDS, 0.1% sodium pyrophosphate) and bands subsequently visualised by autoradiography. 10 × SSC is 1.5 M sodium chloride 150 mM sodium citrate.

*Immunohistochemistry and* in situ *hybridisation*

Sections for immunohistochemistry were cut at 3 μm, dewaxed in xylene and rehydrated through graded alcohols. Slides were washed several times in buffer (100 mM Tris, 0.1% Tween 20, 5% normal goat serum) before overnight incubation with rabbit polyclonal P450 antisera (diluted 1:50 in buffer), washed (3 × 10 min) and incubated (30 min) with biotinylated goat anti-rabbit IgG (1:500 in buffer). Sections were then exposed to streptavidin-peroxidase conjugate (Dako, Ltd. UK) for 30 min and then developed with 3,3′ diaminobenzidene and lightly counterstained with haematoxylin.

*In situ* hybridisation was performed essentially according to Herrington *et al.* (1990), with the following modifications: Formalin fixed sections were dewaxed and then permeabilised in 0.2 M HCl followed by 0.3% Triton-X100 in phosphate-buffered saline. Proteinase K was used at 10–20 mg ml$^{-1}$ and sections were postfixed in 0.4% paraformaldehyde. After 1 h at 37°C in prehybridisation buffer (0.6 M NaCl, 10% dextran sulfate, 30% deionised formamide, 0.1% sodium pyrophosphate, 0.2% polyvinylpyrrolidone, 0.2% Ficoll), 20–40 ng of biotinylated probe was incubated overnight at 37°C. Detection of oligonucleotide binding was achieved using a mouse anti-biotin antibody, followed by alkaline phosphatase labelling using nitroblue tetrazolium (NBT)/5-bromo-4-chloroindolyl phosphate (BCIP), overnight at 4°C for visualisation. The oligonucleotides used were an 18mer from Exon 2 (bp 112–130) of the cDNA sequence of the human CYP2B6 gene, (5′CCCATATTTCTCTCGGAA 3′ antisense); (5′TTCCGAGAGAAATATGGG 3′ sense). These oligonucleotides had 6 mismatches relative to the mouse Cyp2b9 sequence. Three biotin molecules were added at the 5′ end of each oligonucleotide using a monomer developed by Link Technologies, Cumbernauld, Scotland.

### Results

A variety of compounds, known to induce P450s from a range of gene families or subfamilies in animal models, was administered to mice bearing either human breast or colon tumours as xenografts. The effectiveness of the induction protocol was confirmed by demonstrating that the predicted changes in murine hepatic cytochrome P450 gene expression had occurred (data not shown).

Control untreated xenograft samples had extremely low P450 content. However, a protein which reacted with the antibody to CYP2A1 was identified by Western blot analysis (Figure 1). This protein had a different mobility to recombinant human CYP2A6, which has been associated with hepatic coumarin hydroxylase activity (Miles *et al.*, 1990; Yamano *et al.*, 1990). Very low levels of proteins reacting with antibodies to CYP2B1 and CYP2C6 were detected in the colon tumour using the highly sensitive ECL detection system (not shown). The relative mobility of the protein detected with the CYP2B1 antibody (54.5 kD) was different to human CYP2B6 (51.0 kD). The mobility of the protein detected with anti CYP2C6 was the same as human CYP2C8 (54.5 kD).

Tumour cytochrome P450 content was significantly altered by the administration of several of the P450-inducing agents tested (Figure 1). 3-Methylcholanthrene (3-MC) and β-naphthoflavone (β-NF) are both well characterised inducers of P450 proteins in the CYP1A and CYP2A gene families (Guengerich, 1987). Both of these compounds could induce the level of a CYP1A protein, probably CYP1A1, within both the colon and breast tumour tissues. The level of a protein reacting with the CYP2A1 antibody (Figure 1, lower band) was also increased 2–3-fold and stronger upper band approximately 5-fold in the breast and colon samples respectively, by both 3-MC and β-NF. A third protein with a higher apparent molecular weight (55.5 kD), was also slightly induced by these compounds in the breast, but not the colon tissue. Interestingly, in further experiments using ECL as the detection system, 3-MC also caused slight induction (2–3-fold) in the level of the protein reacting with the antibody to CYP2B1 (data not shown).

TCPOBOP and dexamethasone are potent 'phenobarbital-like' inducing agents in the mouse, but have virtually no effect on the expression of the major phenobarbital-inducible isozymes in the rat (Poland *et al.*, 1980; Meehan *et al.*, 1988). The ability of these two compounds to induce human tumour P450 levels was compared to the effects of phenobarbital.

TCPOBOP, at a dose of 3 mg kg$^{-1}$ was a potent modulator of human P450 expression, inducing proteins in the CYP2A, CYP2B, CYP2C, CYP3A and CYP4A gene families. In breast tissue, the constitutively expressed CYP2A form

**Figure 1** Induction of cytochrome P450 expression in human breast and colon tumour xenografts. Human colon **a**, or breast **b**, tumours were grown as xenografts in immune deficient mice to a diameter of 1 cm. Animals were then treated intra-peritoneally with a variety of compounds, as described in Materials and methods. Microsomal fractions were prepared from tumour samples and analysed for cytochrome P450 content by Western blotting using the antibodies shown in the left hand track. Fifteen μg of microsomal protein was loaded per track. Following transfer to nitrocellulose, antibody incubations were carried out as described previously (Meehan *et al.*, 1988), using $^{125}$I-Protein A for visualisation. C = control, MC = 3-methylcholanthrene, NF = β-naphthoflavone, Cl = clofibric acid, T = TCPOBOP, D = dexamethasone, PB = phenobarbital, S = purified rat P450 standards to the CYP1A2, CYP2A1, CYP2B1, CYP2C6, CYP3A4 and CYP4A1 isozymes respectively. HLM is a human liver microsomal sample. The relative mobility of recombinant human P450s are shown in the right hand track and were aligned from a blot where the liver and tumour samples were run on the same gel.

(lower band) and that slightly induced by 3-MC (mr 555 kD) were both markedly induced. In colon tissue, only the major 51.5 kD protein (band 2) was induced. Proteins reacting with antisera to CYP2B1 were markedly increased on TCPOBOP treatment in both the breast and colon tissues, with the induction of CYP2B in the colon being particularly pronounced. This induced protein had the same mobility as the constitutively expressed form and appears to be distinct from CYP2B6. Whether this protein is CYP2B7 or a novel cytochrome P450 isozyme is currently being investigated. Proteins reacting with the CYP2C6 and CYP3A1 antibodies were also induced in the xenograft tissue by TCPOBOP. Based on mobilities of these induced proteins they may be CYP2C8 and CYP3A4 respectively. However, further work is required to clarify this. Perhaps the most surprising effect of TCPOBOP was the profound increase in the expression of a protein reacting with the antibody to CYP4A1. This induction was particularly pronounced in the breast tumour, but was also observed in the colon samples (data not shown).

In a similar manner to TCPOBOP, dexamethasone also induced proteins in the CYP2B gene family in both the breast and colon tumours (Figure 1) but did not increase the levels of P450's in the CYP3A gene family (Figure 1 and not shown). Interestingly, this compound also caused a marked induction of the protein reacting with the CYP4A antibody in colon but not in breast tissue. Relative to TCPOBOP and dexamethasone, phenobarbital was much less effective as an inducing agent in the xenograft tumours, but did cause some increase in the levels of CYP2A and CYP2B proteins in both tumours and CYP4A in the breast xenograft. Clofibric acid had very little effect on tumour P450 expression but did appear to induce the protein reacting with the CYP2A antibody (lower band). This compound did not induce proteins reacting with the antibody to CYP4A1.

*Localisation of the induced proteins*

A potential problem in the use of tumour xenografts to study human gene regulation is the possibility that the observed changes in gene expression are due to infiltration of the tumour with cells of the host and that the enzymes studied are, in fact, murine. Immunohistochemical studies with P450 polyclonal antisera unequivocally demonstrated that the induced proteins are localised within malignant human epithelial cells rather than mouse derived tissues (Figure 2). The cells staining positive with the antisera were morphologically malignant on histopathological examination. Tumour cells were derived from a multiply passaged cell line and no significant murine stroma was visible (A.H. Wyllie, personal communication). Staining of P450 proteins was often found to be heterogeneous, with some areas of tumour staining strongly, while others were weak or even negative. Good agreement was obtained between the levels of protein detected on Western blot analysis and immunohistochemical staining.

*Evidence that induction of CYP2B protein expression by TCPOBOP occurs at the level of transcription*

CYP2B mRNA levels were determined in the human colon xenograft by Northern blot analysis using a cDNA probe for human CYP2B6 (Figure 3). Two bands of 3.0 kb and 1.65 kb respectively were detected in both the human liver mRNA and the xenograft samples. Cyp2b mRNA isolated from the liver of the host mouse had a much smaller transcript size (1.5 kb), and was clearly distinguishable from the mRNA species identified in the tumour tissue. It is interesting to note that the predominant mRNA species induced in the tumour tissue is the 3.0 kb transcript, while that in the liver runs at 1.65 kb. This supports the possibility that the major inducible P450 isozyme within the tumour is distinct from CYP2B6, but is from the same gene family.

CYP2B mRNA from both the liver of the host mouse and the associated xenograft tumour was highly induced on xenobiotic treatment. In the liver, induction by TCPOBOP was far greater than that observed on 3-methylcholanthrene treatment. Within the tumour tissue, however, mRNA levels for both the 3-methylcholanthrene and TCPOBOP treated groups were equally induced.

The induction of a CYP2B protein within the xenograft tumours was confirmed by *in situ* hybridisation for mRNA using an oligonucleotide probe derived from the human CYP2B6 cDNA sequence (Figure 4). This oligonucleotide has 6 mismatches with mouse Cyp2b9, and would not be expected to hybridise to Cyp2b mRNA. These studies confirmed the localisation of P450 mRNA to the human breast and colon tumour cells and, in agreement with the Northern blot analysis, indicated that the observed induction of CYP2B protein had occurred at the level of transcription.

**Figure 2** Immunohistochemical localisation of cytochrome P450 proteins in control and TCPOBOP-treated animals. P450 expression in xenograft colon **a**, and breast **b**, tumours was determined using antibodies against rat (i) CYP1A2 (reactive with both CYP1A1 and CYP1A2) (ii) CYP2B1 and (iii) CYP4A1 proteins. Experimental details were as described in Materials and methods. Control tumours showed low or negative expression except for breast which expressed CYP2B at low levels and colon which demonstrated focal reactivity for CYP1A. Treated breast tumours demonstrated strong reactivity for all three antibodies. Induction of CYP4A was less marked in colon and the expression of CYP1A in treated colon tumour remained very heterogeneous.

**Figure 3** Analysis of CYP2B mRNA levels in a xenograft colon tumour Northern blot anlysis of mRNA levels was as described in Materials and methods. Animals were treated intraperitoneally with either vehicle alone, or with 3-MC or TCPOBOP. HL = human liver RNA, C = Control xenograft, or xenografts from MC = 3-methylcholanthrene (100 mg $kg^{-1}$), $TC_1$ = TCPOBOP (3 mg $kg^{-1}$) or $TC_2$ = TCPOBOP (15 mg $kg^{-1}$) treated animals.

**Figure 4** Detection of CYP2B mRNA in the tumour xenografts by *in situ* hybridisation. *In situ* hybridisation was carried out using oligonucleotide probes derived from Exon 2 of the human CYP2B6 gene, as described in Materials and methods. **a**, Breast xenograft from control animals using the antisense oligonucleotide probe showing little significant labelling; **b**, TCPOBOP-treated breast xenograft using antisense probe showing widespread, but heterogeneous cytoplasmic reactivity; **c**, TCPOBOP-treated colon xenograft using a sense probe, showing no specific binding; **d**, TCPOBOP treated colon xenograft using antisense probe showing strong, fairly homogeneous cytoplasmic reactivity.

### Discussion

A model is described which can be applied to study how environmental and hormonal factors regulate the expression of human cytochrome P450 enzymes. The strength of this model is that it circumvents the previously intractable problem that the expression and induction of cytochrome P450 expression in cell lines is lost, possibly due to methylation of the cytochrome P450 genes (Antequara *et al.*, 1990). It also allows humoral or metabolic factors such as the effects of hormones and lymphokines which can not be easily studied in *in vitro* systems to be evaluated.

Although the influence of the mouse host on the experimental results cannot be ruled out, the observed changes in gene expression were clearly seen within the human tumour tissues and therefore reflect the responsiveness of human cells to the administered agents. Both the tumour tissues studied maintained their human phenotype and are characteristic of the tumour tissue of origin.

The data presented here demonstrate that human breast and colon tumours constitutively express low levels of certain cytochrome P450 isozymes. This is in agreement with previous findings which demonstrate that P450 expression is detectable in tumour biopsy samples from these tissues (Senler *et al.*, 1985; Forrester *et al.*, 1990; Murray *et al.*, 1991). The constitutive expression of a protein in the CYP2A gene family in both the breast and colon samples is interesting in view of the recent report of the isolation of a CYP2A protein from mouse liver tumours (Maurice *et al.*, 1991).

Evidence is presented that cytochrome P450 levels in human colon and breast tumour tissues can be influenced by environmental agents. The patterns of gene regulation observed were often consistent with what is known about hepatic P450 regulation in rats and mice. These data imply that human breast and colon tissues have the capacity to express P450s from many of the gene families involved in foreign compound metabolism. Some of the proteins identified in the xenograft tissues had different mobilities on SDS-PAGE from the human hepatic P450s. Whether these proteins represent novel enzymes is currently being investigated.

Of particular interest in this study was the capacity of dexamethasone and TCPOBOP to induce human tumour P450 expression. The marked species specificity in the capacity of these compounds to induce CYP2B and CYP2C proteins raised the question of whether they would also be active in man. Both compounds however, especially TCPOBOP, had marked effects on the expression of many human cytochrome P450 isozymes, particularly in the induction of CYP2B and CYP4A proteins. Immunohistochemistry and *in situ* hybridisation studies demonstrated that the induction of CYP2B occurred within human tissue and indicated that it occurred either at the level of transcription or message stabilisation.

It is conceivable that the species-specific effects of TCPOBOP are due to effects on another tissue such as the pituitary and that this compound may still be inactive in man. This is unlikely to be the case however, as we have shown that TCPOBOP-mediated P450 induction in the liver is due to direct action on hepatocytes (Smith *et al.*, 1992). Species differences in the metabolism of TCPOBOP to the active inducer could also explain the specificity of this compound. However, this also seems unlikely as TCPOBOP is an inert compound, and there is no evidence that metabolism is required before it exerts its inductive effects (Poland *et al.*, 1980). The immunohistochemical data unequivocally confirms that TCPOBOP has the capacity to alter human gene expression.

The profound induction of CYP4A proteins in the human breast tumour by TCPOBOP and dexamethasone, and to a lesser extent by phenobarbital, is intriguing. There are currently no reports on CYP4A expression in either colon or breast tissue. The metabolic consequences of CYP4A regulation in these tissues, the expression of which is associated with peroxisome proliferation and fatty acid metabolism, remains to be elucidated.

Mechanisms of regulation of human P450s in extra-hepatic tissues, by agents other than polycyclic aromatic hydrocarbons (McLemore et al., 1990; Vang et al., 1991; Stralka & Strobel, 1991), are essentially unknown. The xenograft model allows questions relating to this previously intractable problem to be addressed. Although animal models have previously shown that colon tissue will respond to compounds such as phenobarbital (Degawa et al., 1991; Hammond & Strobel, 1990), the particular P450 isozymes affected are poorly characterised. A similar situation exists for rodent mammary tissues. It remains to be established how closely P450 regulation within tumour tissues reflects that of the normal tissue of origin. It has been demonstrated, however, that tumour cell lines, as well as rat hepatic preneoplastic lesions, retain many of the transcription factors required for P450 induction (Buchmann et al., 1987; Vang et al., 1991), the loss of expression being possibly due to DNA methylation (Antequara et al., 1990).

Finally, these studies demonstrate that exogenous agents have the capacity to alter human tumour cytochrome P450 levels. This has a variety of implications. Firstly, individuality in the expression of tumour P450 levels may relate to the responsiveness of patients to chemotherapeutic agents which are metabolised by this enzyme system. Secondly, the ability to modulate tumour P450 levels raises the possibility that the efficacy of chemotherapeutic agents which require P450-mediated activation in order to exert their anti-tumour effects could be increased by prior or concomitant administration of drugs which induce intra-tumour P450 expression. Of particular interest in this regard are cyclophosphamide, Tamoxifen and the novel morpholinodoxorubicin derivatives. These compounds require bioactivation by specific cytochrome P450 isozymes (Powis & Prough, 1987; Jalacot et al., 1991; Mani & Kupfer, 1991; Lewis et al., 1992) which we found inducible within the human tumours by TCPOBOP.

We are grateful to Dr F.R. Balkwill for access to the xenograft tumours and to Hazel Holdsworth for the maintenance and treatment of the xenograft mice. Part of this work was supported by a grant from Scottish Hospitals Endowment Research Trust (SHERT 1056).

**Abbreviations:**

TCPOBOP, 1,4 bis 2-(3,5 dichloropyridyloxy)benzene; β-NF, β-naphthoflavone; 3-MC, 3-methylcholanthrene; P450, cytochrome P450; ECL, enhanced chemiluminescence.

**References**

ANTEQUARA, F., BOYES, J. & BIRD, A. (1990). High levels of *de novo* methylation and altered chromatin structure at CpG islands in cell lines. *Cell*, **62**, 503–514.

BERGER, D.P., WINTERHALTER, B.R. & FIEBIG, H.H. (1991). In *The Nude Mouse in Oncology Research*, Boren, B. & Winograd, B. (eds), pp. 165–1884 CRC Press: Boca Raton.

BUCHMANN, A., SCHWARTZ, M., SCHMIDT, R., KUHLMANN, W.D., WOLF, C.R. & OESCH, F. (1987). Expression and inducibility of drug metabolising enzymes in preneoplastic and neoplastic lesions of rat liver during nitrosamine-induced hepatocarcinogenesis. *Arch. Toxicol.*, **60**, 198–203.

CONNEY, A.H. (1982). Induction of microsomal enzymes by foreign chemicals and carcinogenesis by polycyclic aromatic hydrocarbons: G.H.A. Clowes memorial lecture. *Cancer Res.*, **42**, 4875–4917.

DEGAWA, M., MIURA, S. & HASHIMOTO, Y. (1991). Expression and induction of cytochrome P450 isozymes in hyperplastic nodules of rat liver. *Carcinogenesis*, **12**, 2151–2156.

FALZON, M., MCMAHON, J.B., SCHULLER, H.M. & BOYD, M.R. (1986). Metabolic activation and cytotoxicity of 4-ipomeanol in human non-small cell lung cancer lines. *Cancer Res.*, **46**, 3484–3489.

FORRESTER, L.M., HAYES, J.D., MILLIS, R., BARNES, D., HARRIS, A.L., SCHLAGER, J.J., POWIS, G. & WOLF, C.R. (1990). Expression of glutathione S-transferase and cytochrome P450 in normal and tumour breast tissue. *Carcinogenesis*, **11**, 2163–2170.

FORRESTER, L.M., HENDERSON, C.J., GLANCEY, M.G., BACK, D.J., PARK, B.K., BALL, S.E., KITTERINGHAM, N.R., MCLAREN, A.W., MILES, J.S., SKETT, P. & WOLF, C.R. (1992). Relative expression of cytochrome P450 isoenzymes in human liver and association with the metabolism of drugs and xenobiotics. *Biochem. J.*, **281**, 359–368.

GUENGERICH, F.P. (ed.) (1987). *Mammalian Cytochrome P450*, Vols. I & II, CRC Press: Boca Raton.

HAMMOND, D.K. & STROBEL, H.W. (1990). Human colon tumor cell line LS174T drug metabolising system. *Mol. Cell Biochem.*, **93**, 95–105.

HERRINGTON, C.S., FLANNERY, D.M.J., MCGEE, J.O'D. (1990). In *In Situ Hybridisation*, Polak, J.M. & McGee, J.O'D. (eds). Oxford University Press: Oxford.

JALACOT, F., SIMON, I., DREANO, Y., BEAUNE, P., RIOCHE, C. & BERTHON, F. (1991). Identification of the cytochrome P450IIIA family of the enzymes involved in the N-demethylation of tamoxifen in human liver microsomes. *Biochem. Pharmacol.*, **41**, 1911–1919.

LEWIS, A.D., HICKSON, I.D., ROBSON, C.N., HARRIS, A.L., HAYES, J.D., GRIFFITHS, S.A., MANSON, M.M., HALL, A.E., MOSS, J.E. & WOLF, C.R. (1988). Amplification and increased expression of alpha class glutathione S-transferase encoding genes associated with resistance to nitrogen mustards. *Proc. Natl Acad. Sci. USA*, **85**, 8511–8515.

LEWIS, A.D., LAU, D.H.M., DURAN, G.E., WOLF, C.R. & SIKIC, B.I. (1992). Role of cytochrome P450 from the human CYP3A gene family in the potentiation of the toxicity of morpholino doxorubicin by human liver microsomes. *Cancer Res.*, (in press).

LINDBERG, W.J., SCHUETZ, E.G., REDFORD, K.S. & GUZELIAN, P.S. (1991). Hepatocellular phenotype *in vitro* is influenced by biophysical features of the collagenous substratum. *Hepatology*, **13**, 282–288.

MALIK, S.T.A., GRIFFIN, D.B., FIERS, W. & BALKWILL, F.R. (1989). Paradoxical effects of tumour necrosis factor in experimental ovarian carcinoma. *Int. J. Cancer*, **44**, 918–925.

MANI, C. & KUPFER, D. (1991). Cytochrome P-450-mediated activation and irreversible binding of the antioestrogen tamoxifen to proteins in rat and human liver: Possible involvement of flavin-containing monooxygenases in tamoxifen metabolism. *Cancer Res.*, **51**, 6052–6058.

MAURICE, M., EMILIANI, S., DALET, B.I., DERANCOURT, J. & LANGE, R. (1991). Isolation and characterisation of a cytochrome P-450 of the IIA subfamily from human liver microsomes. *Eur. J. Biochem.*, **200**, 511–517.

MCLEMORE, T.L., ADELBERG, S., LIU, M.C., MCMAHON, N.A., YU, S.J., HUBBARD, W.C., CZERWINSKI, M., WOOD, T.G., STORENG, R., LUBET, R.A., EGGLESTON, J.C., BOYD, M.R. & HINES, R.N. (1990). Expression of CYP1A1 gene in patients with lung cancer: Evidence for cigarette smoke-induced gene expression in normal lung tissue and for altered gene regulation in primary pulmonary carcinomas. *J. Natl Cancer Inst.*, **82**, 1333–1339.

MEEHAN, R.R., FORRESTER, L.M., STEVENSON, K., HASTIE, N.D., BUCNMANN, A., KUNZ, H.W. & WOLF, C.R. (1988). Regulation of phenobarbital-inducible cytochrome P450s in rat and mouse following dexamethasone administration and hypophysectomy. *Biochem. J.*, **254**, 789–797.

MILES, J.S., MCLAREN, A.W., FORRESTER, L.M., GLANCEY, M.G. LANG, M.A. & WOLF, C.R. (1990). Identification of the human liver cytochrome P450 responsible for coumarin 7-hydroxylase activity. *Biochem. J.*, **267**, 365–371.

MURRAY, G.I., FOSTER, C.O., BARNES, T.S., WEAVER, R.J., SNYDER, C.P., EWEN, S.W.B., MELVIN, W.T. & BURKE, M.D. (1991). Expression of cytochrome P450IA in breast cancer. *Br. J. Cancer*, **63**, 1021–1023.

NEBERT, D.W. & GONZALEZ, F.J. (1987). P450 genes: Structure, evolution and regulation. *Annual Rev. in Biochem.*, **56**, 945–993.

NEBERT, D.W., NELSON, D.R., COON, M.J., ESTABROOK, R.W., FEYEREISEN, R., FUJII-KURIYAMA, Y., GONZALEZ, F.J., GUENGERICH, F.P., GUNSALUS, I.C., JOHNSON, E.F., LOPER, J.C., SATO, R., WATERMAN, M.R. & WAXMAN, D.J. (1991). The P450 superfamily: Update on new sequences, gene mapping and recommended nomenclature. *DNA & Cell Biol.*, **10**, 1–14.

OMENN, G.S. & GELBOIN, H.V. (eds) (1984). In: *Genetic Variability in Responses to Chemical Exposure*, Banbury Report **16**, 3, Cold Spring Harbor Laboratories: Cold Spring Harbor, New York.

PAINE, A.J. (1990). The maintainance of cytochrome P-450 inrat hepatocyte culture: some applications of liver cell cultures in the study of drug metabolism, toxicity and induction of the P-450 system. *Chem. Biol. Interact.*, **74**, 1-31.

PASENEN, M., STACEY, S., LYKKESFELDT, A., BRIAND, P., HINES, R. & AUTRUP, H. (1988). Induction of cytochrome P4501A1 gene expression in human breast tumour cell lines. *Chem. Biol. Interact.*, **66**, 223-232.

POLAND, A., MAK, I., GLOVER, E., BOATMAN, R.J., EBETINO, F.H. & KENDE, A.S. (1980). 1,4-bis[2-(3,5-dichloropyridyloxy)]benzene, a potent phenobarbital-like inducer of microsomal monooxygenase activity. *Mol. Pharmacol.*, **18**, 571-580.

POWIS, G. & PROUGH, R.A. (eds). (1987). In: *Metabolism and Action of Anticancer Drugs*, Taylor & Francis: London.

SENLER, T.I., DEAN, W.L., MURRAY, L.F. & WITTLIFF, J.L. (1985). Quantification of cytochrome P-450-dependent cyclohexane hydroxylase activity in normal and neoplastic reproductive tissues. *Biochem. J.*, **227**, 379-387.

SMITH, G., HENDERSON, C.J., PARKER, M.G., WHITE, R., BARS, R.G. & WOLF, C.R. (1993). 1,4-bis[2-(3,5-dichloropyridyloxy)] benzene, an extremely potent modulator of murine hepatic cytochrome P450 gene expression. *Biochem. J.*, **289**, 807-813.

STANLEY, L.A., CARMICHAEL, J. & WOLF, C.R. (1992). Cytochrome P-450 induction in human lung tumour-derived cell lines: Characterisation and effects of inflammatory mediators. *Eur. J. Biochem.*, **208**, 521-529.

STRALKA, D. & STROBEL, H.W. (1991). Characterisation of cytochrome P450-dependent dimethylhydrazine metabolism in human colon microsomes. *Cancer*, **68**, 2363-2369.

TOWBIN, H., STAEHLIN, T. & GORDON, J. (1979). Electrophoretic transfer of proteins from polyacrylamide gels to nitrocellulose sheets: procedure and some applications. *Proc. Natl Acad. Sci. USA*, **76**, 4350-4354.

VANG, O., JENSEN, H. & AUTRUP, H. (1991). Induction of cytochrome P-450 IA1, IA2, IIB1, IIB2 and IIE1 by broccoli in rat liver and colon. *Chem. Biol. Interact.*, **78**, 85-96.

WOLF, C.R. (1991). Metabolic factors in cancer susceptibility. *Cancer Surv.*, **9**, 437-474.

YAMANO, S., TATSUNO, J. & GONZALEZ, F.J. (1990). The CYP2A3 gene product catalyzes coumarin 7-hydroxylation in human liver microsomes. *Biochemistry*, **29**, 1322-1329.